# The role of parenting styles and depression in predicting suicidal ideation vulnerability among university students

**DOI:** 10.1186/s12912-025-03307-2

**Published:** 2025-06-20

**Authors:** Mai El-Ghareap Hassan, Soma Ibrahim Ali, Amal Sobhy Mahmoud, Fatma Elemam Hafez, Ateya Megahed Ibrahim

**Affiliations:** 1 Family and Community Health Nursing, Faculty of Nursing, Port Said University, Port Said City, Egypt; 2 Psychiatric Nursing and Mental Health Department, Faculty of Nursing, Port Said University, Port Said City, Egypt; 3 Mental Health Nursing, College of Nursing, Gulf Medical University, Ajman, UAE; 4 Psychiatric Nursing and Mental Health, Faculty of Nursing, Port Said University, Port Said City, Egypt; 5 Psychiatric Nursing and Mental Health, Faculty of Nursing, Misr University for Science and Technology (MUST), October City, Egypt; 6 Community Health Nursing, Faculty of Nursing, Mansoura University, Mansoura, Egypt; 7 College of Nursing, Prince Sattam Bin Abdualziz University, Alkarj, Saudi Arabia

**Keywords:** Parenting styles, Depression, Suicidal ideation, University students

## Abstract

**Background:**

Parenting practices are relevant in shaping children’s psychological development, and positive parenting tends to be associated with positive outcomes. These practices significantly affect adolescents’ mental health by influencing depression, suicidal behaviours, and attitudes towards suicide.

**Aim:**

To examine the predictive role of parenting styles and depression in vulnerability to suicidal ideation among university students.

**Methods:**

A descriptive correlational study was conducted with 480 university students recruited using stratified multistage cluster sampling from the Faculty of Health Sciences (Medicine and Nursing), Mathematical Sciences (Engineering and Computer Science), and Human Sciences (Arts and Commerce) at Port-Said University. The instruments used for data collection included the Parenting Styles and Dimensions Questionnaire (PSDQ), Beck Depression Inventory, Morey Suicidal Ideation Scale (SUI), and a sociodemographic data sheet. Statistical analysis included Spearman correlation, non-parametric tests, and mediation analysis to explore the direct and indirect relationships.

**Results:**

Participants’ ratings ranged from moderate levels of depression (13.85 ± 7.68) to suicidal ideation (10.20 ± 5.32). In this regard, participants provided higher ratings for the scores of authoritative parenting reported by both mothers and fathers, with mean scores of 48.61 ± 10.59 and 45.96 ± 10.34, respectively. Suicidal ideation was somewhat negatively related to parenting style (*p* < 0.001), and there was a negative relationship between depression and the maternal parenting style (*p* < 0.0001). In contrast, depression was positively correlated with suicidal ideation (*p* < 0.01).

**Conclusion:**

The study concluded that Parenting styles significantly influence university students’ mental health. Warmth combined with structure in parenting, which is authoritative parenting, is associated with lower levels of depression and suicidal ideation. These findings suggest that promoting positive parenting practices may foster students’ mental well-being.

**Clinical trial:**

No clinical trial.

## Introduction

Suicide is a global public health problem that affects more than 700,000 individuals worldwide. Over 77% of global suicides occurred in low- and middle-income countries in 2019. In 2021, suicide was the third leading cause of death among 15–29-year-olds globally, with 73% of all suicides occurring in low- and middle-income countries [1]. Suicidal ideation is the most sensitive predictor of suicide attempts and actions and a significant indicator of mental health problems [2]. Suicidal ideation (SI), often known as suicidal thoughts or ideas, is a general term that encompasses various thoughts, desires, and obsessions related to suicide and death. While some scholars view suicide planning as a distinct stage, others refer to it as a discussion [3]. According to Wang et al. [4], the concept or action of lowering life expectations does not necessarily result in bodily injury.

Evidence suggests that a complex interplay between environmental and personal factors may lead to the development of suicidal ideation or attempts [5]. Genetics and home environments have been identified as the main causes of suicidal thoughts in adolescents and young people. Rather than being biological in nature, it leads to adoption behaviours, including mood disorders, impulsive and violent behaviour [6, 7].

Parenting significantly affects mental health. According to a study of 604 Portuguese teenagers, parenting style influences the development of secure attachment and supports the hypothesis that parental attachment plays a mediating role in the relationship between parenting style and suicidal thoughts [8]. Three general categories can be used to describe parenting styles: permissive, authoritarian, and authoritative. Authoritative parenting is characterized by high levels of warmth, consistency, and open communication. However, low levels of demandingness and discipline and high levels of responsiveness to a child’s wants and preferences are characteristics of the permissive parenting style.

In contrast, the authoritarian approach is characterized by high consistency and low warmth, both of which have unique effects on a child’s development [9, 10]. Parental bonding is one of the key elements influencing parent-child relationships and family closeness [11]. In addition to intellectual, social, and emotional development, parenting styles can impact the mental health of children and adolescents [12–16]. Researchers have discovered a correlation between parenting styles and depression levels in adults, adolescents, and children [17].

Previous study indicates that university students subjected to dysfunctional parenting, characterized by excessive control and minimal affection, are at an increased risk of developing mental health issues. The ‘affectionless control’ style, for example, which is characterised by high protection and little care, has been associated with increased depression and suicidal thoughts in medical students [18]. Similarly, Tugnoli et al. showed that adolescents who experience neglectful parenting, a sign of a lack of emotional support, are more likely to experience depression. These findings underscore the significant impact of parenting styles on the mental health of university students [19].

One of the most prevalent mood disorders is depression, which frequently causes a loss of interest and happiness, as well as confusion and hopelessness [20]. Teenage depression and suicide are major mental health issues in all societies. There is also a concern that teenage depression may portend a lifetime of struggle with mental illness [21, 22]. As depression is the most prevalent mental illness among those who die by suicide, it has been identified as a significant risk factor for suicide [23, 24].

### Significance of the study

Adolescence is a risky period accompanied by emotional, cognitive, and behavioural changes, during which the likelihood of the onset of mental disorders is significantly higher [25]. Globally, one in seven individuals aged 10–19 years has some form of mental disorder. Suicide is the third leading cause of death among individuals aged 15–29 years [1]. Suicide attempts surge during adolescence, and depression underlies this trend [26, 27].

According to the World Health Organization, 62% of adolescents with depression experience severe suicidal ideation and behaviours [28]. Parental patterns encountered during childhood are responsible for the determinants of an individual’s psycho-development and the risk of mental disorders. Negative parenting, particularly authoritarian and neglectful styles, has been found to significantly elevate suicidal behaviours, whereas authoritative styles have a protective effect [29]. In addition, negative parenting, mainly by fathers, is related to an increased risk of suicidal ideation (SI) and suicide attempts (SA) [30, 31]. Adolescents who had experienced authoritarian parenting supported suicide more than those who experienced democratic or permissive parenting [32], while supportive parenting was less related to adolescent psychopathology and non-suicidal self-injury [33].

Depression, characterized by feelings of helplessness, hopelessness, and emotional distress, has consistently emerged as the strongest mediating factor linking parenting styles to suicidal ideation. Suicidal ideation was considered at length, along with depressive symptoms. The literature indicates a strong association between suicidal ideation and suicide attempts among individuals with lower levels of depression [34]. Teenagers who reported suicidal thoughts had significantly higher depression scores [35], and suicide-related outcomes were especially prevalent among females and youth displaying depressive symptoms [36].

The COVID-19 pandemic has intensified concerns regarding adolescent depression and suicide risk [37]. Various studies have identified anxiety and depression as key predictors of suicide attempts among teenage self-harmers [38]. Thus, the relationship between parenting styles and suicidal ideation is intricate and influenced by mediating factors such as depression and anxiety. Research has confirmed that negative parenting styles contribute to suicidal ideation through emotional mediators [39, 40]. Consequently, a comprehensive understanding of how parenting styles interact with depression is vital for developing effective interventions targeting university students, ultimately enhancing their mental well-being and reducing suicide risks.

### Theoretical framework

The interaction between parenting styles, depression, and suicidal ideation is supported by several disparate theoretical frameworks in psychology, namely attachment theory and Beck’s cognitive theory of depression. As laid out in Baumrind’s framework for parenting styles, authoritative, authoritarian, permissive, and neglectful parenting styles have a certain effect on the emotional lives of children [9]. Authoritative parenting, characterized by warmth and control, is likely to foster psychological resilience, but both authoritative and neglectful parenting styles are associated with greater emotional distress and dysfunctional coping styles [41].

Insecurely attached individuals, owing to inconsistent or punitive parenting, may elicit cognitive distortions for themselves or other people, a core component of Beck’s depression model [42]. Such distortions, worthlessness, or hopelessness are core antecedents of suicidal ideation. Moreover, the interpersonal theory of suicide [43] proposes that suicidal ideation is a consequence of perceived burdensomeness and thwarted belongingness, both of which may stem from a dysfunctional parental home life and a lack of parental support.

Thus, parenting styles indirectly influence suicidal ideation by regulating an individual’s emotional state, constructing a self-concept, and affecting depression sensitivity. Under the conditions of university life, where students are exposed to high levels of stress and less parental monitoring, the internalized impacts of early parenting are critical predictors of mental health outcomes for specific individuals [44, 45]. The theoretical framework justifies the investigation of parenting styles and depression for their importance in predicting suicidal ideation among university students.

### Aim of the study

- To examine the predictive role of parenting styles and depression in vulnerability to suicidal ideation among university students.

#### Objectives of the study

- Identify different parenting styles among university students.
- Assess the levels of depression among university students.
- Identifying university students’ vulnerability to suicidal ideation.
- Exploring the association between university students’ sociodemographic characteristics, parenting styles, depression levels, and suicidal ideation vulnerability.
- To examine whether parenting styles and depression predict suicidal ideation among university students.

#### Study hypotheses

- H1: There is a significant relationship between parenting styles and vulnerability to suicidal ideation among university students.
- H2: University students with higher depression levels are more likely to experience suicidal ideation.
- H3: The impact of parenting style on suicidal ideation is mediated by the level of depression in university students.

## Methods

### Study design and setting

A descriptive correlational design was employed. This study was conducted at Port Said University, Port Said Governorate, Egypt. The faculties were classified into various disciplines, including health sciences (Medicine and Nursing), mathematical sciences (Engineering and Computer Sciences), and human sciences (Arts and Commerce).

### Study participants

The study targeted university students enrolled in the previously mentioned faculties allied with Port Said University during the academic year 2023–2024. Participants in this study were selected based on the following inclusion criteria: individuals aged 18 years and older, of both genders, who consented to participate, and who were free from mental or physical disabilities.

### Sample size and sampling technique

The sample size was calculated to assess depression rates of 10% or higher [28] and suicidal ideation rates of 14% or higher [46] among university students, with a 95% confidence level, 2% absolute precision, and a design effect of 1.5 for multi-stage cluster sampling. Using the Open Epi software package for a sample size of a single proportion, the required sample sizes were 322 and 450, respectively. Thus, a larger sample size was chosen and increased to 480 to compensate for a non-response rate of approximately 5%.

A stratified multistage cluster sampling approach was used to maintain statistical efficiency and guarantee a wide representation of the university students. The sampling process was carried out in three stages.

### Stages I and II (Stratification and faculty Selection)

The initial level of stratification was based on academic disciplines, dividing faculties into three categories: health, mathematical sciences, and human sciences. This approach was adopted to ensure representation from diverse academic disciplines, as academic backgrounds may influence psychological and social factors, such as parenting styles and mental health indicators [47].

In the second stage, two faculties from each stratum were randomly chosen to form clusters. Random cluster selection is an efficient strategy for working with geographically or administratively grouped population. It also reduces selection bias and ensures variety within each academic domain [48]. The randomly selected faculties included the following:

- Health Sciences: Faculty of Medicine (*N* = 1268), Faculty of Nursing (*N* = 322).
- Mathematical Sciences: Faculty of Engineering (*N* = 2710), Faculty of Computer Sciences (*N* = 668).
- Human Sciences: Faculty of Arts (*N* = 4299), Faculty of Commerce (*N* = 8440).

### Stage III (Student selection and equal Allocation)

The third stage entailed systematic random sampling to recruit students from the chosen faculties. Regardless of the size of the faculty, a predefined number of 80 students were selected. Although this does not reflect proportional sampling based on population size, the fixed sample approach was purposefully used to provide balanced group sizes for subgroup analysis and to adjust for potential confounding factors due to faculty size. The equal allocation technique is typically used when the goal is statistical efficiency and comparability between groups [49, 50]. The emphasis was also on inter-group differences rather than population-representative prevalence [51].

### Sample size justification

The total sample size was calculated to be adequate to detect a correlation coefficient of 0.25 (small effect size) between important study variables (e.g., parenting styles, depression, and suicidal ideation) at a 99% level of significance and 95% study power. Given the sensitivity of the outcomes tested, this high level of precision is crucial when applying standard methods for sample size estimation in correlational studies [52, 53].

### Instruments for data collection

Four instruments were used to gather the data for this study.

### Tool I: A structured demographic data sheet of students and their parents

This structured sheet was developed by researchers in Arabic to assess the personal characteristics of both students and their parents. It comprises the following three sections.

#### Section 1

personal characteristics such as students’ age, sex, faculty name, marital status, grade, and birth order.

#### Section 2

It included data related to parents’ personal characteristics, such as age, marital status, educational level, and job status.

#### Section 3

This includes data related to family, such as the presence of psychiatric illness, kinship relationships, and income.

### Tool II: parenting styles and dimensions questionnaire (PSDQ)

Robinson et al. [54] developed this questionnaire to assess several parenting aspects as well as broader parenting styles. The PSDQ consists of 32 items that assess three parenting styles: authoritative parenting (15 items), authoritarian parenting (12 items), and permissive parenting (five items). Parents must indicate how frequently they display specific behaviours towards their children. Each item in the PSDQ is rated on a 5-point Likert-type scale ranging from 1 (never) to 5 (always).

Scoring was used to categories parents into one of three parenting styles: authoritative (15 items; score range: 0–75), authoritarian (12 items; score range: 0–60), and permissive (five items; score range: 0–25). The mean score for each parenting style determined each parent’s particular style, with the highest mean score placed in one of the three categories, where higher scores indicated more frequent use of the described behaviour. The total scoring system for each parenting style was as follows: none < 30, mild from 30 to 36, moderate from 37 to 43, and high from 44 to 50. The tool has good internal consistency, with Cronbach’s alpha coefficients of 0.86, 0.82, and 0.64 for the authoritative, authoritarian, and permissive subscales, respectively [54].

The Arabic version of the Parenting Styles and Dimensions Questionnaire (PSDQ) was used, which was culturally adapted and validated by Abdelwahab et al. [55]. The adaptation process followed typical protocols, such as forward and backward translation, expert review, and pilot testing among Arabic-speaking students to guarantee clarity and cultural relevance. The tool revealed high internal consistency, with a Cronbach’s alpha of 0.963, indicating its suitability for use in Arabic-speaking communities.

### Tool III: Beck depression inventory (BDI)

This questionnaire has been widely used to detect depression in the general population. It was developed by Beck et al. [56] based on the Diagnostic and Statistical Manual of Mental Disorders (DSM) criteria in English and translated into Arabic by Abdel-Khalek [57]. The questionnaire consisted of 21 items scale that identified the cognitive, behavioural, affective, and somatic symptoms of depression. Each item comprises four statements that rate symptom intensity from 0 to 3. The score for each of the twenty-one questions by counting the numbers to the right of each question. The highest possible score was 63, and the lowest was zero. A total score of 1–10 is considered normal, mild mood disturbances 11–16, borderline clinical depression from 17 to 20, moderate depression 21–30, severe depression 31–40, and scores over 40 are considered extreme depression. For the Arabic version of the Beck Depression Inventory, Cronbach’s α values confirmed prominent scale consistency.

The Arabic version of the Beck Depression Inventory (BDI) was used. This was previously validated by Abdel-Khalek [57]. The cultural adaptation procedure included translation, back-translation, and expert evaluation to ensure linguistic and conceptual consistency with Beck et al.‘s original tool. With a correlation coefficient of.96, the Arabic and English versions showed strong consistency.

### Tool V: Morey suicidal ideation scale (SUI)

It was developed by Morey [58] to measure the frequency and severity of suicidal ideation and plans. It is a self-report scale consisting of 12-items. The SUI scoring system was scored on a four-point Likert scale ranging from zero to three as, not true at all (0), rarely true (1), sometimes true (2), and true nearly all of the time (3). There were two reverse questions (Questions 10 and 12). The total score ranged from 0 to 36, with higher scores indicating higher levels of suicidal ideation. The cut-off point of the scale was less than 60%, indicating a low level of suicidal ideation; 60 to 70% indicated a high level of suicidal ideation; and more than 85% indicated that the case was serious or dangerous.

The tool was translated and culturally adapted according to standard procedures, such as forward and backward translation, expert panel review for semantic and conceptual equivalence, and pilot testing with Arabic-speaking university students to assess clarity and cultural relevance. El Salamony et al. [59], created a version with strong psychometric qualities, including high internal consistency (Cronbach’s alpha > 0.85), and proved construct validity in Arab groups.

### Pilot study

Prior to the commencement of the main study, a pilot study was conducted on 48 randomly chosen university students, comprising 10% of the total number of students in the study. The purpose of the pilot study was to evaluate the clarity and applicability of the study instruments, as well as the amount of time required to complete them. The pilot study results indicated that no adjustments were necessary. Students who participated in the pilot study were excluded from the primary study sample.

### Actual procedure

Initially, the faculties at Port-Said University were grouped into three main areas: human, mathematical, and health sciences. Two faculties were randomly selected from each group for the interviews. Once the purpose of the study was explained, official permission was obtained from six randomly selected faculties taken into consideration. For each faculty, the research team visited the faculty dean’s office to explain the study’s goal and arrange the best day to gather data. To select students from each faculty list, the researchers employed a systematic random selection.

Prior to administering the data collection tools, the research team conducted interviews with students in each faculty who satisfied the eligibility requirements and provided their verbal informed consent after outlining the study’s goal and gaining their cooperation. Students in the study filled out research forms while the researcher’s team was present to respond to any questions that the students had. The researchers assured that the comments would be used exclusively for research purposes and that all information would be kept confidential. The completion of the questionnaires took roughly thirty to forty-five minutes. The time that the students willingly gave was also acknowledged. Data collection will take place over three months, from mid-August 2024 to mid-November 2024.

### Ethical considerations and informed consent

This study was conducted in accordance with the ethical guidelines of the 2013 Declaration of Helsinki. The Scientific Research Ethics Committee of the Faculty of Nursing, Port Said University, Egypt, granted ethical approval for this study under code number NUR (4/8/2024) (40). Consent was obtained from the relevant authorities. Although written consent is typically preferred, verbal consent was used due to cultural norms and institutional practices in the university setting. Given the low risk and non-invasive nature of the study and the cultural context of collecting data within the university, verbal consent was appropriate. Verbal consent was obtained from the students, and the participants were informed of their freedom to withdraw from the study at any time before completion, with no consequences. All participants were fully informed of the goals, risks, and voluntary nature of their involvement in the study. Given the sensitive nature of suicidal ideation, information on mental health support resources was provided to the participants, and the researchers affirmed that all data obtained would be used exclusively for research purposes.

### Statistical analysis

All statistical analyses were performed using IBM SPSS software (version 20.0; IBM Corp., Armonk, NY, USA). Qualitative data were expressed in terms of frequencies and percentages, whereas quantitative measures for central tendency and dispersion were expressed in terms of range, means, and standard deviations. The normal distribution of the data for continuous variables was investigated using the Kolmogorov-Smirnov test. As some variables did not demonstrate a normal distribution, non-parametric tests were used for all statistical analyses.

Two independent groups were compared using the Mann-Whitney U test, while the Kruskal–Wallis test was used to compare the ranks among more than two groups. Spearman’s rank correlation coefficient was applied to test associations between no normally distributed quantitative variables, considering p-values of less than 0.05 statistically significant throughout the analyses in all two-tailed tests used.

Path analysis was used to verify the proposed hypothesis which is “depression mediated the relationship between perceived parenting style and suicidal ideation”. This model examined both direct and indirect effects within a mediation framework. In this model, path “a” assessed the influence of parenting style (maternal or paternal) on depression (mediator), while path “b” assessed the influence of depression on suicidal ideation (outcome), and path “c” evaluated the direct effect of parenting style on suicidal ideation, independent of depression. The effects of “c” encompass the sum of both direct and indirect pathways. Mediation was established if an indirect effect (a × b) was significant and the 95% confidence interval did not include zero. The coefficients for each path (b), t-values, and p-values were reported to test whether each relationship was strong and statistically significant. This approach can provide more insight into the effect of parenting styles on suicidal ideation, at least partly through depression.

## Results

The study sample included 480 students divided equally by year of study and faculty. A gender imbalance was seen, with females representing 74% of respondents. Most participants were first- and second-birth order, and 84.2% had never failed an exam. Parental age indicated that most of the mothers and fathers were between the age of 40–55 and that fathers had more years of education. The most common family composition consisted of 5–6 members, and 80.6% of the participants reported that they had sufficient family income. The sample predominantly comprised individuals residing in urban neighborhoods (80.2%), indicative of a socioeconomically stable urban population (Table 1).

**Table 1:** Distribution of the studied cases according to demographic data (*n* = 480)

Demographic data	No.	%
Faculty		
Faculty of Computer Science	80	16.7
Faculty of Commerce	80	16.7
Faculty of Specific Education	80	16.7
Faculty of Nursing	80	16.7
Faculty of Medicine	80	16.7
Faculty of Engineering	80	16.7
**Year/Level**		
First	120	25
Second	120	25
Third	120	25
Fourth	120	25
**Gender**		
Male	125	26
Female	355	74
**The student’s birth order**		
First	161	33.5
Second	174	36.3
Third	87	18.1
Fourth	31	6.5
Only child	9	1.9
Other	18	3.8
**Have you ever failed an exam before**		
Yes	76	15.8
No	404	84.2
**Mother’s age in years**		
40–45	200	41.7
46–50	138	28.8
51–55	86	17.9
56–60	50	10.4
61–65	6	1.3
**Father’s age in years**		
40–45	32	6.7
46–50	122	25.4
51–55	148	30.8
56–60	108	22.5
61–65	70	14.6
**Mother’s level of education**		
Illiterate (cannot read or write)	28	5.8
Literate (can read and write)	41	8.5
Basic education	23	4.8
Intermediate qualification	232	48.3
University degree	148	30.8
Postgraduate studies	8	1.7
**Father’s level of education**		
Illiterate (cannot read or write)	21	4.4
Literate (can read and write)	31	6.5
Basic education	35	7.3
Intermediate qualification	223	46.5
University degree	160	33.3
Postgraduate studies	10	2.1
**Mother’ s job**		
Employed	220	45.8
Unemployed	260	54.2
**Father’s job**		
Employed	428	89.2
Unemployed	52	10.8
**Mother’s marital status**		
Married	428	89.2
Separated	11	2.3
Divorced	11	2.3
Widowed	30	6.3
**Father’s marital status**		
Married	436	90.8
Separated	9	1.9
Divorced	8	1.7
Widowed	4	0.8
**Are there any mental illnesses in the family?**	(***n* = 480)**	
Yes	43	9
No	437	91
**Number of family member**		
3–4	120	25
5–6	303	63.1
Other	57	11.9
**Income**		
Sufficient	387	80.6
Insufficient	61	12.7
More than enough	32	6.7
**Residence**		
Urban	385	80.2
Rural	95	19.8

The mean depression score was 13.85 ± 7.68, indicating mild depression. The mean score for suicidal ideation was 10.20 ± 5.32, suggesting that the majority of students experienced suicidal thoughts. Mothers were perceived as slightly more authoritative than fathers, with mean scores of 48.61 and 45.96, respectively. In terms of authoritarian parenting, both parents were comparable, although fathers scored marginally higher than mothers, with scores of 40.26 and 39.26, respectively. Both mothers and fathers exhibited the lowest scores for permissive parenting, with fathers scoring lower than mothers. These findings suggest mild psychological distress among the students, with authoritative and authoritarian parenting styles being the most prevalent (Table 2).

**Table 2:** Distribution of the studied cases according to depression, suicidal ideation and parenting styles and dimensions questionnaire (PSDQ) (*n* = 480)

Variables		
**Depression**	Total Score (0–63)	13.85 ± 7.68
	% Score	21.98 ± 12.20
**Suicidal ideation**	Total Score (0–36)	10.20 ± 5.32
	% Score	28.32 ± 14.77
**Parenting styles and dimensions questionnaire (PSDQ)**	**Mother**	**Father**
	**Mean ± SD**	**Mean ± SD**
**Authoritative**		
Total Score	48.61 ± 10.59	45.96 ± 10.34
% Score	56.02 ± 17.65	51.60 ± 17.23
**Authoritarian**		
Total Score	39.26 ± 6.85	40.26 ± 6.70
% Score	56.79 ± 14.26	58.87 ± 13.95
**Permissive**		
Total Score	14.55 ± 3.13	13.81 ± 3.54
% Score	47.76 ± 15.63	44.07 ± 17.68
**Overall parenting styles ** **(32 − 160)**		
Total Score	**102.42 ± 12.38**	**100.03 ± 11.90**
% Score	**55.02 ± 9.67**	**53.15** **9.30**

Strong positive associations were found between suicidal ideation and depression (rs = 0.231, *p* < 0.001), with higher depression levels being associated with more suicidal ideation. The study identified negative correlations between maternal parenting and both depression (rs = -0.194, *p* < 0.001) and suicidal ideation (rs = -0.306, *p* < 0.001), indicating that increased maternal parenting is associated with reduced levels of depression and suicidal ideation. Furthermore, paternal parenting also demonstrated negative associations with depression (rs = -0.143, *p* = 0.002) and suicidal ideation (rs = -0.230, *p* < 0.001); however, these associations were less pronounced compared to those observed with maternal parenting. This suggests that nurturant parenting styles protect against depression and suicidal ideation (Table 3).

**Table 3:** Correlation between different parameters

		Depression	Suicidal ideation	Parenting styles and dimensions questionnaire (PSDQ)	
				Mother	Father
Depression	**r**_**s**_	1.000	0.231^*^	-0.194^*^	-0.143
	**p**		< 0.001^*^	< 0.001^*^	0.002
Suicidal ideation	**r**_**s**_		1.000	-0.306^*^	-0.230^*^
	**p**			< 0.001^*^	< 0.001^*^
Parenting styles and dimensions (PSDQ)					
Mother	**r**_**s**_			1.000	0.435^*^
	**p**				< 0.001^*^
Father	**r**_**s**_				1.000
	**p**				

**r**_**s**_: **Spearman coefficient;** *: Statistically significant at *p* ≤ 0.05

There were significant differences in depression scores based on faculty (*p* = 0.002) and academic years (*p* < 0.001), where the depression scores were found to be higher among Medicine and Commerce students, as well as second academic year students. There was high variability in suicidal ideation scores by faculty (*p* < 0.001), previous exam failure (*p* < 0.001), fathers’ age (*p* = 0.009), and mothers’ employment status (*p* = 0.034). This finding demonstrates that academic stress and familial factors, particularly parental employment, contribute to  positive mental health outcomes (Table 4).

**Table 4:** Relation between total score for depression and suicidal ideation with demographic data (*n* = 480)

	*N*	Total score for Depression		Total score for suicidal ideation	
		Mean ± SD.	Median	Mean ± SD.	Median
**Faculty**					
Faculty of Computer Science	**80**	11.93 ± 6.16	11	13.05 ± 4.58	13
Faculty of Commerce	**80**	14.66 ± 4.26	14.5	11.53 ± 4.61	11
Faculty of Specific Education	**80**	13.56 ± 4.29	13	11.39 ± 4.55	11
Faculty of Nursing	**80**	13.86 ± 4.25	13	7.66 ± 3.44	7
Faculty of Medicine	**80**	16.34 ± 11.23	13	8.14 ± 5.76	6.5
Faculty of Engineering	**80**	12.73 ± 11.27	11	9.41 ± 6.36	6
**H (p)**		19.321^*^(0.002^*^)		88.613^*^(< 0.001^*^)	
**Year/Level**					
First	**120**	11.40 ± 6.17	11	10.02 ± 4.74	9
Second	**120**	16.91 ± 8.53	16	10.38 ± 5.23	9
Third	**120**	13.15 ± 7.60	14	10.39 ± 4.84	10.5
Fourth	**120**	13.93 ± 7.28	13	9.99 ± 6.36	8
**H (p)**		40.782^*^(< 0.001^*^)		**2.627** **(0.453)**	
**Gender**					
Male	**125**	13.94 ± 7.87	13	10.86 ± 5.78	10
Female	**355**	13.81 ± 7.63	13	9.96 ± 5.13	9
**U (p)**		**21868.50 (0.811)**		**20517.00 (0.209)**	
**The student’s birth order**					
First	**161**	14.36 ± 8.24	13	10.25 ± 5.27	10
Second	**174**	13.06 ± 7.25	12	9.79 ± 5.40	8.5
Third	**87**	13.21 ± 7.23	13	10.67 ± 5.43	10
Fourth	**31**	15.45 ± 8.18	13	10.16 ± 5.52	9
Only child	**9**	13.78 ± 4.09	12	10.11 ± 4.51	11
Other	**18**	17.17 ± 8.47	15.5	11.44 ± 4.66	11.5
**H (p)**		**6.203 (0.287)**		**4.776 (0.444)**	
**Have you ever failed an exam before**					
Yes	**76**	14.80 ± 7.16	14	13.58 ± 5.61	13
No	**404**	13.67 ± 7.77	13	9.56 ± 5.02	9
**U (p)**		**13635.50 (0.121)**		**8714.0** ^*^ **(< 0.001** ^*^ **)**	
**Mother’s age in years**					
40–45	**200**	14.59 ± 8.35	13	9.97 ± 5.11	9
46–50	**138**	13.06 ± 6.41	12	10.44 ± 5.38	9
51–55	**86**	13.81 ± 7.77	13	10.59 ± 6.17	10
56–60	**50**	12.98 ± 6.92	12	9.86 ± 4.43	9
61–65	**6**	15.0 ± 14.45	14.5	9.0 ± 4.86	6.5
**H (p)**		**3.437 (0.487)**		**1.234 (0.872)**	
**Father’s age in years**					
40–45	**32**	15.34 ± 7.67	14.5	10.13 ± 4.28	9
46–50	**122**	14.89 ± 7.78	13	10.74 ± 5.54	10
51–55	**148**	13.49 ± 7.69	13	9.28 ± 5.13	8
56–60	**108**	12.60 ± 6.64	12	11.27 ± 5.04	11.5
61–65	**70**	14.01 ± 8.77	12	9.57 ± 5.85	8
**H (p)**		**7.019(0.135)**		**13.569** ^*^ **(0.009** ^*^ **)**	
**Mother’s level of education**					
Illiterate (cannot read or write)	**28**	16.36 ± 10.47	14	10.86 ± 5.69	10
Literate (can read and write)	**41**	14.44 ± 8.45	13	9.46 ± 4.13	9
Basic education	**23**	14.30 ± 8.59	13	10.61 ± 5.57	8
Intermediate qualification	**232**	12.92 ± 6.93	12	10.22 ± 5.45	9
University degree	**148**	14.57 ± 7.91	14	10.24 ± 5.37	10
Postgraduate studies	**8**	14.13 ± 2.80	14	8.88 ± 4.45	6.5
**H (p)**		**6.567 (0.255)**		**1.678 (0.892)**	
**Father’s level of education**					
Illiterate (cannot read or write)	**21**	14.0 ± 9.93	12	11.90 ± 6.53	12
Literate (can read and write)	**31**	14.16 ± 6.16	13	9.58 ± 4.40	8
Basic education	**35**	11.66 ± 4.63	12	9.86 ± 4.83	9
Intermediate qualification	**223**	13.37 ± 7.42	12	10.09 ± 5.29	9
University degree	**160**	14.84 ± 8.52	13.5	10.46 ± 5.48	10
Postgraduate studies	**10**	14.80 ± 5.53	16	7.80 ± 4.52	7
**H (p)**		**5.924 (0.314)**		**4.021 (0.546)**	
**Mother’ s job**					
Employed	**220**	14.35 ± 7.65	13.5	10.73 ± 5.55	10
Unemployed	**260**	13.42 ± 7.70	12.5	9.74 ± 5.08	9
**U (p)**		**26602.0 (0.186)**		**25393.0 (0.034** ^*^ **)**	
**Father’s job**					
Employed	**428**	13.84 ± 7.79	13	10.23 ± 5.42	9
Unemployed	**52**	13.90 ± 6.78	12	9.92 ± 4.40	10
**U (p)**		**10981.00 (0.876)**		**11169.0 (0.965)**	
**Mother’s marital status**					
Married	**428**	13.93 ± 7.75	13	10.22 ± 5.41	9
Separated	**11**	12.18 ± 5.90	11	8.91 ± 4.46	8
Divorced	**11**	15.18 ± 9.24	16	11.91 ± 4.95	11
Widowed	**30**	12.83 ± 6.82	13	9.73 ± 4.34	9
**H (p)**		**0.856 (0.836)**		**2.086 (0.555)**	
**Father’s marital status**					
Married	**436**	13.92 ± 7.74	13	10.27 ± 5.42	9
Separated	**9**	12.0 ± 4.74	10	9.56 ± 5.32	8
Divorced	**8**	15.25 ± 9.77	11.5	9.75 ± 3.96	9.5
Widowed	**4**	13.0 ± 1.63	13	12.25 ± 5.38	12
**H (p)**		**0.761 (0.859)**		**0.792 (0.851)**	
**Are there any mental illnesses in the family?**					
Yes	**43**	13.35 ± 3.94	13	9.93 ± 5.65	8
No	**437**	13.89 ± 7.96	13	10.22 ± 5.29	9
**U (p)**		**9168.50 (0.793)**		**9829.50 (0.616)**	
**Number of family member**					
3–4	**120**	12.95 ± 6.42	13	10.62 ± 5.12	9.5
5–6	**303**	14.07 ± 8.38	13	9.81 ± 5.35	9
other	**57**	14.54 ± 5.98	15	11.35 ± 5.40	10
**H (p)**		**3.064 (0.216)**		**5.758 (0.056)**	
**Income**					
Sufficient	**387**	13.60 ± 7.51	13	10.19 ± 5.25	9
Insufficient	**61**	15.38 ± 8.50	14	10.20 ± 5.18	11
More than enough	**32**	13.91 ± 8.09	13.5	10.28 ± 6.47	9
**H (p)**		**2.076 (0.354)**		**0.148 (0.929)**	
**Residence**					
Urban	**385**	13.57 ± 7.14	13	10.19 ± 4.95	9
Rural	**95**	14.97 ± 9.54	14	10.20 ± 6.62	8
**U (p)**		**19585.50 (0.283)**		**17051.50 (0.306)**	

**SD: Standard deviation U: Mann Whitney test H: H for Kruskal Wallis test**

p: p value for comparing between different categories

*: Statistically significant at *p* ≤ 0.05

Maternal and paternal parenting style scores showed a statistically significant variance among faculties, with nursing and engineering students viewing their mothers through a more authoritative lens, while nursing, specific education, and medicine students rated their fathers’ parenting styles higher. Parenting style scores also varied according to academic year; second-year students had the lowest scores for both mothers (*p* = 0.008) and fathers (*p* = 0.018). Students who had never failed an exam had significantly higher maternal parenting scores (*p* = 0.011), suggesting that supportive parenting might be a factor related to academic success. Maternal scores were higher among students from rural areas (*p* = 0.014), whereas paternal scores were higher among unemployed mothers (*p* = 0.029). This indicates that the academic year, family dynamics, and geographical location could influence students’ perceptions of their parents’ parenting styles (Table 5).

**Table 5:** Relation between total score for parenting styles and dimensions questionnaire (PSDQ) with demographic data (*n* = 480)

	*N*	Parenting styles and dimensions questionnaire (PSDQ)			
		Mother		Father	
		Mean ± SD.	Median	Mean ± SD.	Median
**Faculty**					
Faculty of Computer Science	**80**	97.08 ± 8.64	96.5	98.89 ± 8.71	99
Faculty of Commerce	**80**	95.59 ± 7.65	96	96.51 ± 7.65	97
Faculty of Specific Education	**80**	99.76 ± 10.97	97	98.53 ± 10.76	100.5
Faculty of Nursing	**80**	112.48 ± 8.13	113	106.96 ± 9.37	106.5
Faculty of Medicine	**80**	101.53 ± 15.17	101.5	99.11 ± 15.54	100.5
Faculty of Engineering	**80**	108.11 ± 12.67	108	100.19 ± 14.61	98
**H (p)**		**126.950** ^*^ **(< 0.001** ^*^ **)**		**43.713** ^*^ **(< 0.001** ^*^ **)**	
**Year/Level**					
First	**120**	104.79 ± 10.52	104	101.74 ± 10.41	103
Second	**120**	99.58 ± 11.64	99	96.97 ± 13.50	98.5
Third	**120**	103.70 ± 13.61	102	101.92 ± 11.55	102
Fourth	**120**	101.63 ± 13.01	100	99.50 ± 11.39	100
**H (p)**		**11.889** ^*^ **(0.008** ^*^ **)**		**10.037** ^*^ **(0.018** ^*^ **)**	
**Gender**					
Male	**125**	101.75 ± 12.36	100	100.61 ± 10.45	101
Female	**355**	102.66 ± 12.40	101	99.83 ± 12.38	100
**U (p)**		**23166.0 (0.463)**		**21289.0 (0.500)**	
**The student’s birth order**					
First	**161**	101.91 ± 12.40	100	98.58 ± 11.53	99
Second	**174**	102.40 ± 12.07	100	100.12 ± 11.71	101
Third	**87**	102.47 ± 12.17	101	99.02 ± 12.98	99
Fourth	**31**	102.81 ± 15.55	97	104.03 ± 12.44	105
Only child	**9**	103.67 ± 12.61	102	105.0 ± 10.64	103
Other	**18**	105.72 ± 11.13	108.5	107.61 ± 6.04	106
**H (p)**		**2.708 (0.745)**		**18.833** ^*^ **(0.002** ^*^ **)**	
**Have you ever failed an exam before**					
Yes	**76**	99.08 ± 10.02	97	98.96 ± 9.89	101
No	**404**	103.05 ± 12.69	101	100.23 ± 12.24	100
**U (p)**		**18162.0** ^*^ **(0.011** ^*^ **)**		**16111.0** **(0.494)**	
**Mother’s age in years**					
40–45	**200**	102.25 ± 12.42	100.5	99.60 ± 12.40	101
46–50	**138**	103.52 ± 12.40	101.5	100.75 ± 10.99	100
51–55	**86**	101.67 ± 11.64	100	99.81 ± 11.74	100
56–60	**50**	101.84 ± 13.51	99	100.82 ± 12.82	102
61–65	**6**	98.50 ± 13.25	99.5	94.67 ± 10.97	93
**H (p)**		**1.780 (0.776)**		**1.934 (0.748)**	
**Father’s age in years**					
40–45	**32**	99.00 ± 12.15	98.5	94.97 ± 13.87	97
46–50	**122**	103.33 ± 12.44	102	100.99 ± 11.03	102
51–55	**148**	102.78 ± 12.20	102	101.16 ± 12.28	100
56–60	**108**	101.16 ± 11.83	100	98.44 ± 11.08	99
61–65	**70**	103.61 ± 13.48	100.5	100.74 ± 12.27	102
**H (p)**		**4.577 (0.334)**		**9.395 (0.052)**	
**Mother’s level of education**					
Illiterate (cannot read or write)	**28**	99.43 ± 11.92	97	96.75 ± 14.97	97
Literate (can read and write)	**41**	104.00 ± 14.12	107	98.15 ± 12.56	101
Basic education	**23**	102.22 ± 8.98	102	100.87 ± 11.70	102
Intermediate qualification	**232**	103.47 ± 12.39	101	100.60 ± 11.21	100.5
University degree	**148**	101.20 ± 12.48	99	100.34 ± 12.23	100
Postgraduate studies	**8**	97.63 ± 8.18	96.5	96.38 ± 10.47	95
**H (p)**		**1.678 (0.181)**		**3.059 (0.691)**	
**Father’s level of education**					
Illiterate (cannot read or write)	**21**	97.43 ± 13.26	98	95.10 ± 11.67	93
Literate (can read and write)	**31**	101.90 ± 12.52	102	99.68 ± 13.07	103
Basic education	**35**	105.14 ± 11.25	105	99.77 ± 11.69	102
Intermediate qualification	**223**	103.38 ± 12.05	101	100.92 ± 12.05	101
University degree	**160**	101.23 ± 12.83	100	99.38 ± 11.50	99
Postgraduate studies	**10**	102.80 ± 11.98	106	102.90 ± 11.81	101.5
**H (p)**		**6.440 (0.266)**		**5.713 (0.335)**	
**Mother’ s job**					
Employed	**220**	101.91 ± 12.23	100	98.99 ± 12.07	99
Unemployed	**260**	102.86 ± 12.52	102	100.91 ± 11.70	102
**U (p)**		**29978.50 (0.362)**		**31895.50** ^*^ **(0.029** ^*^ **)**	
**Father’ s job**					
Employed	**428**	102.67 ± 12.42	101	100.12 ± 11.66	101
Unemployed	**52**	100.40 ± 12.03	98	99.27 ± 13.79	98.5
**U (p)**		**10028.50 (0.244)**		**10641.50 (0.606)**	
**Mother’s marital status**					
Married	**428**	102.41 ± 12.51	100	100.21 ± 11.96	101
Separated	**11**	102.09 ± 11.38	99	101.55 ± 9.38	99
Divorced	**11**	100.73 ± 10.19	95	100.27 ± 13.37	99
Widowed	**30**	103.30 ± 12.09	104	96.83 ± 11.30	96.5
**H (p)**		**0.819 (0.845)**		**2.202 (0.532)**	
**Father’s marital status**					
Married	**436**	102.30 ± 12.50	100	100.12 ± 11.97	101
Separated	**9**	101.89 ± 10.86	99	100.0 ± 8.80	99
Divorced	**8**	104.38 ± 9.88	103	104.63 ± 11.39	103
Widowed	**4**	101.0 ± 16.87	101.5	100.0 ± 6.22	100
**H (p)**		**0.422 (0.936)**		**0.984 (0.805)**	
**Are there any mental illnesses in the family?**					
Yes	**43**	104.44 ± 11.72	105	101.51 ± 13.19	103
No	**437**	102.22 ± 12.44	100	99.89 ± 11.77	100
**U (p)**		**8295.50 (0.205)**		**8522.0 (0.314)**	
**Number of family member**					
3–4	**120**	100.76 ± 12.77	99	98.93 ± 12.58	99
5–6	**303**	102.77 ± 12.41	101	99.98 ± 11.83	101
Other	**57**	104.07 ± 11.18	104	102.63 ± 10.53	104
**H (p)**		**3.612 (0.164)**		**5.316 (0.070)**	
**Income**					
Sufficient	**387**	102.36 ± 12.48	101	100.36 ± 11.82	101
Insufficient	**61**	102.23 ± 12.09	99	98.92 ± 11.29	100
More than enough	**32**	103.53 ± 12.04	102	98.22 ± 13.98	99
**H (p)**		**0.635 (0.728)**		**1.681 (0.431)**	
**Residence**					
Urban	**385**	101.86 ± 12.37	100	99.66 ± 11.68	99
Rural	**95**	104.72 ± 12.23	104	101.54 ± 12.70	102
**U (p)**		**21263.0** ^*^ **(0.014** ^*^ **)**		**20092.50 (0.136)**	

**SD: Standard deviation U: Mann Whitney test H: H for Kruskal Wallis test**

p: p value for comparing between different categories

*: Statistically significant at *p* ≤ 0.05

**Fig. 1:**
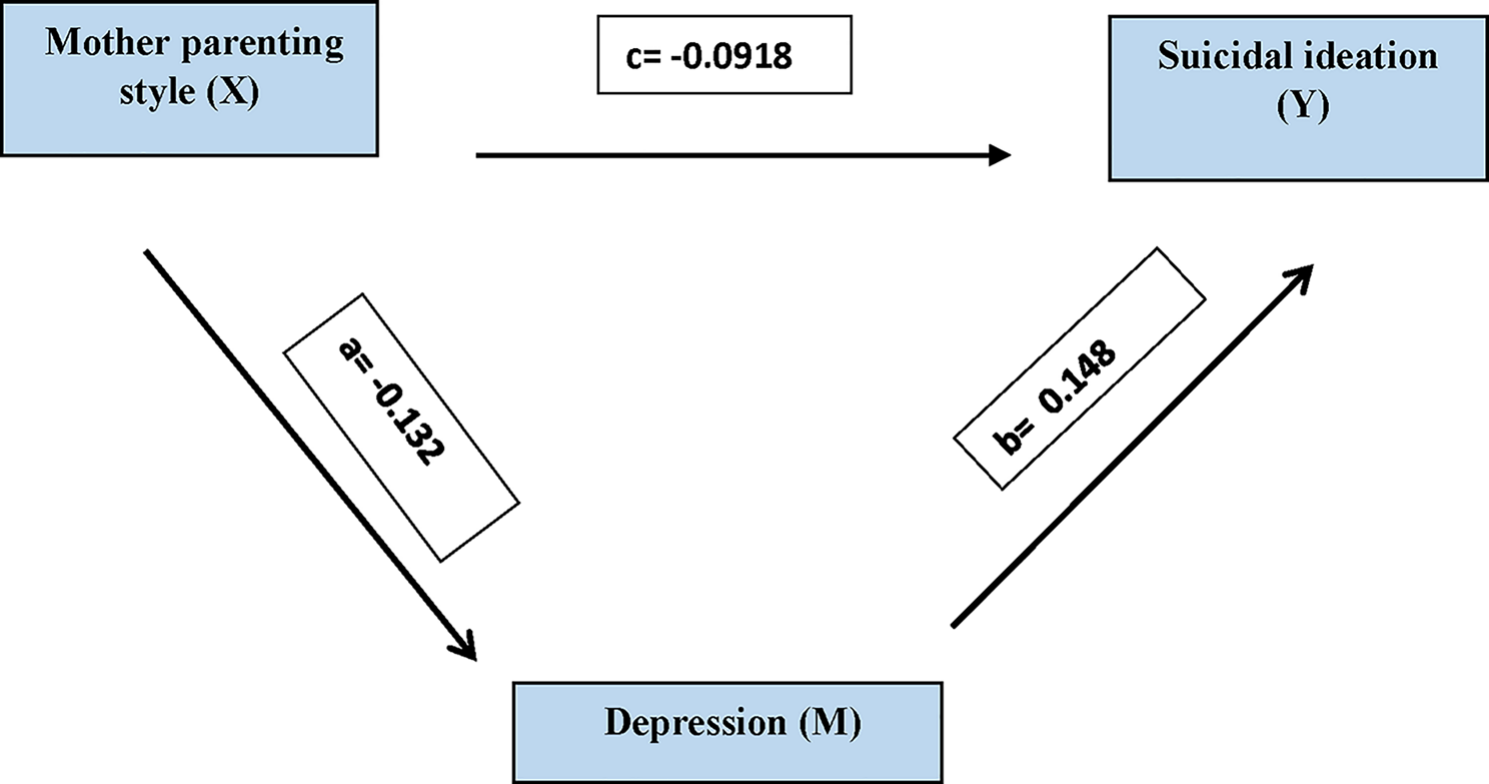
Mediation Analysis Summary: The Impact of Mother Parenting Style on Depression and Suicidal Ideation. Mother parenting style has a significant impact on Depression (b = -0.132, t = 4.774^*^, p < 0.001^*^). This is path a. Mother parenting style has a significant impact on Suicidal ideation (b = -0.092, t = 4.838^*^, p < 0.001^*^). This is a direct effect (c’). Depression also had a significant impact on suicidal ideation (b = 0.148, t = 4.847, *p* < 0.001). This is path b

The mediation analysis found evidence for partial competitive mediation, which was statistically significant: maternal parenting style negatively predicted depression (b = -0.132, t = 4.774, *p* < 0.001), and depression positively predicted suicidal ideation (b = 0.148, t = 4.847, *p* < 0.001). The indirect effect of maternal parenting on suicidal ideation through depression was significant (b = -0.0196), and the 95% confidence interval [–0.0766, − 0.0195] excluded zero, indicating that maternal parenting causes suicidal ideation both directly and indirectly through depression. The direct effect of maternal parenting on suicidal ideation remained significant (b = -0.0918, t = 4.838, *p* < 0.001) even when depression was considered a mediator. These findings show that maternal parenting is important for reducing suicidal ideation, both directly and indirectly, by alleviating depression (Fig. 1; Table 6).

**Table 6:** Mediation pathways of mother parenting style on depression and suicidal ideation

Relationship	Total Effect	Direct Effect	Indirect Effect	95% CI		t–statistics	Conclusion
				LL	UL		
Mother parenting style–> Depression–> Suicidal ideation	-0.1114 (t = 5.8740^*^, *p* < 0.001^*^)	-0.0918 (t = 4.8383 ^*^, *p* < 0.001^*^)	-0.0196	-0.0766	-0.0195	3.130 (Sig.)	**Partial** Mediation Competitive

Mother parenting style has a significant impact on Depression (b = -0.132, t = 4.774^*^, *p* < 0.001^*^). This is path a

Mother parenting style has a significant impact on Suicidal ideation

(b = -0.092, t = 4.838^*^, *p* < 0.001^*^). This is **a direct effect (c’)**

Depression also had a significant impact on suicidal ideation (b = 0.148, t = 4.847, *p* **< 0.001**). This is path b

Indirect Effect was calculated by multiplying indirect effects. a & b

–a (-0.132) * b (0.148) = -0.0196

Direct Effect = -0.0918; Total Effect = Direct Effect (c’) + a*b = -0.1114

**Fig. 2:**
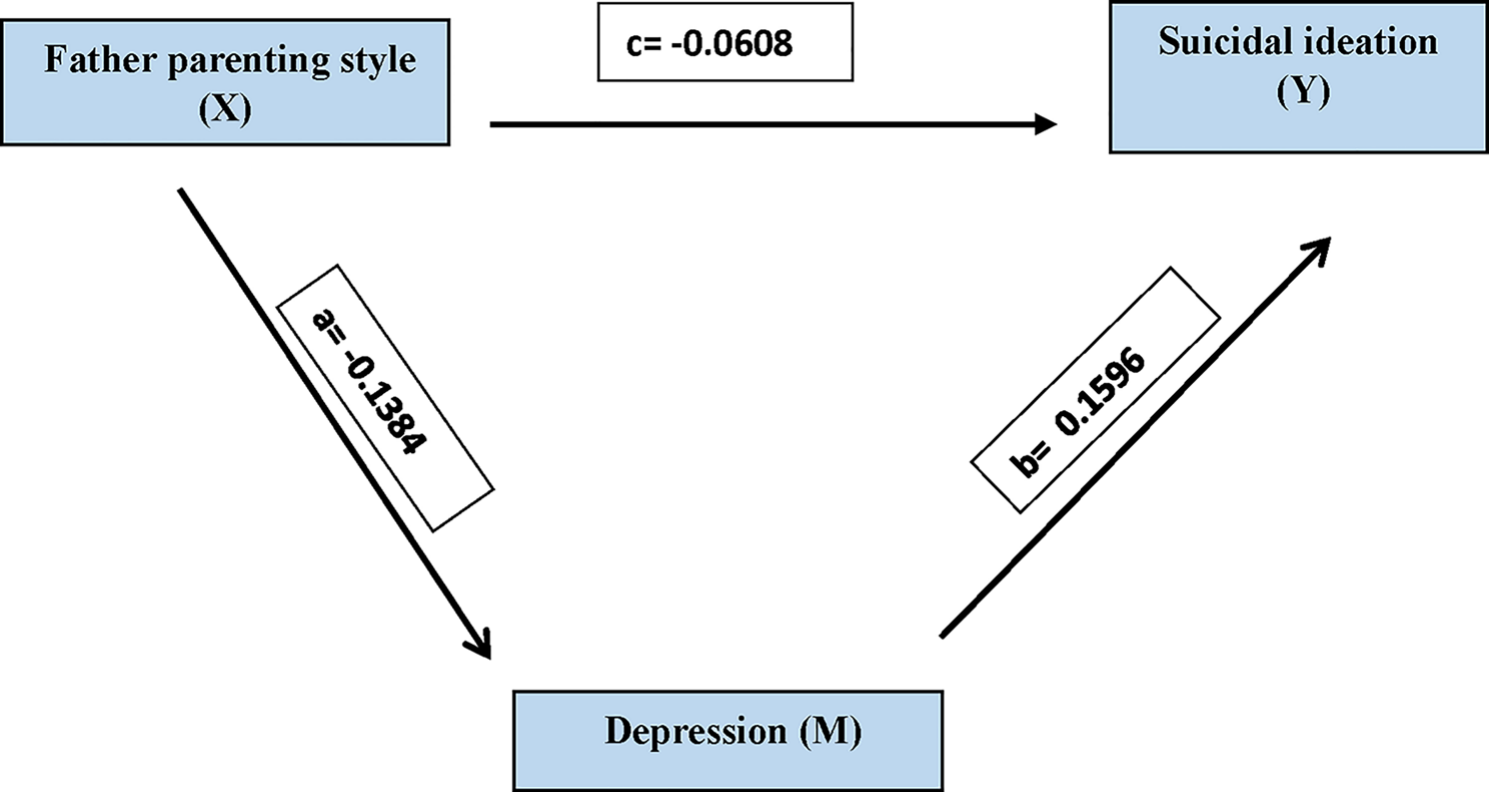
Mediation Model of Father Parenting Style on Depression and Suicidal Ideation. Father parenting style has a significant impact on Depression (b = -0.1384, t = 4.7980^*^, *p* < 0.001^*^). This is path a. Father parenting style has a significant impact on Suicidal ideation (b = -0.0608, t = 3.0338^*^, *p* = 0.0025^*^). This is a direct effect (c’). Depression also had a significant impact on suicidal ideation (b = 0.1596, t = 5.1432, *p* < 0.001). This is path b

Father parenting style had a significant effect on suicidal ideation via depression, with a total effect of -0.0829 (t = 4.1263, *p* < 0.001). The direct effect of paternal parenting style on suicidal ideation was − 0.0608 (t = 3.0338, *p* = 0.0025), and the remaining indirect effect through depression was − 0.0221, with a 95% confidence interval for the indirect effect between − 0.0832 and − 0.0217. This formed the basis for a partial mediation model in which depression mediates the relationship between fathers’ parenting styles and suicidal ideation. Father parenting style adversely affected depression (b = -0.1384, t = 4.7980, *p* < 0.001), which subsequently augmented suicidal ideation (b = 0.1596, t = 5.1432, *p* < 0.001). This illustrates that depression partially mediates the influence of fathers’ parenting styles on suicidal ideation (Fig. 2; Table 7).

**Table 7:** Mediation pathways of father parenting style on depression and suicidal ideation

Relationship	Total effect	Direct effect	Indirect effect	95% CI		t–statistics	Conclusion
				LL	UL		
Father parenting style–> Depression–> Suicidal ideation	-0.0829 (t = 4.1263^*^, *p* < 0.001^*^)	-0.0608 (t = 3.0338 ^*^, *p* = 0.0025^*^)	-0.0221	-0.0832	-0.0217	3.146 (Sig.)	**Partial** Mediation Competitive

Father parenting style has a significant impact on Depression (b = -0.1384, t = 4.7980^*^, *p* < 0.001^*^). This is path a

Father parenting style has a significant impact on Suicidal ideation

(b = -0.0608, t = 3.0338^*^, *p* = 0.0025^*^). This is **a direct effect (c’)**

Depression also had a significant impact on suicidal ideation (b = 0.1596, t = 5.1432, *p* **< 0.001**). This is path b

Indirect Effect was calculated by multiplying indirect effects. a & b

–a (-0.1384) * b (0.1596) = -0.0221

Direct Effect = -0.0608, Total Effect = Direct Effect (c’) + a*b = -0.0829

## Discussion

University students often face a range of challenges that can significantly impact their mental health, including academic pressure, family dynamics, and personal struggles. Among these, mental health issues, such as depression and suicidal ideation, are increasingly prevalent and alarming. Academic stress resulting from the pressure to perform, meet deadlines, and navigate social expectations can exacerbate anxiety and depression in students. In addition, parenting style plays a crucial role in shaping students’ emotional and mental well-being. Research suggests that authoritative parenting, characterised by warmth, support, and structure, can buffer the negative effects of stress and promote better mental health outcomes among university students [60–62].

This complex interplay between academic pressure, family influence, and mental health outcomes has become an important area of research, highlighting the need for universities to address these factors in their mental health support systems. Understanding how these factors interact can provide valuable insights into strategies for mitigating mental health problems among university students and promoting overall well-being [63–65].

This study highlights notable mental health concerns among university students, with findings indicating mild depression and moderate suicidal ideation, suggesting a significant proportion of students may experience psychological distress, warranting attention. This is consistent with a study conducted among university students in UK, which showed that while almost 28% were mildly depressed, 37% had a high suicide risk. This shows that mental health problems among students are common and require more attention and care from universities [66].

The moderate level of suicidal ideation observed in this study suggests that the students may not have reached the critical thresholds of psychological distress typically associated with suicidal behaviour. However, even low levels of suicidal ideation highlight the need for timely mental health interventions, as depression is a known risk factor for self-harm and suicide. These findings are consistent with previous research that has reported higher levels of suicidal ideation among university students. For example, a study in the UK found a significant prevalence of suicidal ideation and associated it with mental health risk factors such as depression and anxiety [67]. Similarly, a mixed-methods study in Egypt identified risk factors, such as female gender, lack of social support, and academic stress, as contributing to higher rates of suicidal behaviour [68], while research in Kenya also found a higher prevalence of suicidal ideation among university students [69].

When exploring parenting styles, students tended to view their mothers as more democratic and supportive, consistent with a largely authoritative parenting style. Fathers were perceived as more authoritarian, suggesting an inclination towards controlling or stricter behaviours. For both parents, the least frequently reported style was permissive parenting. These patterns suggest that parenting styles, particularly the balance of support and control, can influence students’ emotional health and, in some way, their experiences of depression and suicidal ideation.

Parenting styles, particularly authoritative parenting, were significantly associated with lower depression and suicidal ideation. Students who reported experiencing authoritative parenting from both parents had fewer depressive symptoms. These findings suggest that the warmth and structural characteristics of authoritative parenting may play a protective role in the mental health of students. Authoritative parenting, which emphasises open communication, emotional support, and appropriate boundaries, may help students cope with stress more effectively, leading to lower depression and suicidal ideation. In university students, these parenting practices may promote emotional regulation and resilience, reducing vulnerability to mental health problems that are often exacerbated by academic and social stressors.

These findings are consistent with Ibrar et al. [70], who linked authoritative parenting to positive mental health outcomes among university students. Liu et al. [64] also found that supportive parenting helps buffer stress and supports the physical health of Chinese university students. In addition, Yıldız [71], Alt et al. [72], and Hamied et al. [73] suggested that positive parenting practices characteristic of authoritative style improve emotional regulation, which may contribute to the reduced vulnerability to depression among university students observed in this study.

The findings of this study support the large body of existing research by showing that depression plays a central role in the development of suicidal ideation among university students. Depression, a significant risk factor for suicidal ideation, increases the likelihood of suicidal ideation. Students who experience authoritative parenting, which is characterised by warmth and structure, are less likely to experience depressive symptoms, which decreases the likelihood of suicidal ideation. This mediating effect emphasises the importance of both positive parenting and mental health interventions in preventing suicidal behaviour among students.

Several studies [74–78] supported the link between depression and suicidal ideation in college students, emphasising the role of several risk factors, including self-criticism, psychiatric comorbidity, and socioeconomic status. Mediation analysis demonstrated that both maternal and paternal parenting styles significantly influenced depression and suicidal ideation in university students. Higher levels of positive maternal parenting were linked to fewer depressive symptoms and had a direct negative impact on suicidal ideation. Depression was a significant mediator in this relationship, with a notable indirect effect, confirming partial competitive mediation. This finding suggests that maternal parenting affects suicidal ideation both directly and through its influence on depression. These findings emphasise the importance of enhancing maternal support to reduce depression and suicidal thoughts, highlighting the importance of emotional support within university mental health strategies.

Similarly, paternal parenting style was a negative predictor of depression and a direct and indirect predictor of suicidal ideation through depression. Mediation analysis validated the mediating role of depression between paternal support and suicidal ideation, identifying the role of fathers in university students’ mental health. Overall, the results justify the inclusion of maternal and paternal measures in family centered mental health interventions to reduce depression and suicidal ideation among university students.

Several studies support this finding. Fadakar et al. [79] identified depression as a critical factor influencing suicidality among university students in the Eastern Mediterranean region. El Salamony et al. [59] highlighted the importance of emotional regulation and self-control in mitigating suicide risk, further emphasizing depression as a mediating factor. Kabbash et al. [80] reported the substantial impact of mental health issues, particularly depression, on suicidal ideation among Egyptian university students. Chukwuemeka and Obi-Nwosu [81] illustrated the moderating role of emotion regulation in the association between depression and suicidal ideation, thereby supporting the findings of this study. Boyes et al. [82] demonstrated that family functioning and emotion regulation affect mental health outcomes, including self-harm, further reinforcing the role of depression in mediating the effects of family dynamics on suicidal behavior.

Demographic data showed a generally balanced representation of students across faculties and academic years, with a notable proportion of female students. While some studies have suggested that gender may play a role in mental health outcomes [67, 68], the results of this study did not reveal a strong gender difference in mental health outcomes. This may be due to the similar pressures faced by male and female students in academic settings, which are influenced more by academic performance and stressors related to examinations and deadlines. Similarly, the high proportion of students living in urban areas is consistent with trends in other studies, suggesting that urban living may be correlated with greater access to resources, including mental health support [69]. However, the lack of strong demographic effects highlights the complexity of students’ mental health and suggests that factors beyond gender and place of residence may play a greater role in predicting mental health.

The results of the current study showed that mild to moderate levels of depression and occasional suicidal thoughts were present among university students, which could be explained by several demographic factors. The study findings indicated that medical students are more likely to experience depression, which could be explained by the increased demands and pressures of medical schools, which can make them feel overwhelmed and depressed. These findings are supported by studies that show similar trends, such as Kamruzzaman et al. [83] found a significant prevalence of depression, anxiety, and stress among Bangladeshi students. Attia [84] highlighted similar issues in Egypt. Beshr et al. [85] reported high rates among Yemeni medical students, and studies in Qatar, Lebanon, and Al-Quds University showed comparable mental health challenges among students. These studies highlight the widespread mental health challenges faced by university students worldwide.

Moreover, depression scores differed significantly across academic years, with second-year students showing the highest levels. This finding supports the idea that depression is influenced by specific stressors during university life, such as the challenges of transitioning to more advanced coursework or personal responsibilities. Suicidal ideation is consistent across academic years, suggesting that the risk is not tied to specific stages but rather to general stressors affecting all students. This result is consistent with a survey conducted among medical students in Saudi Arabia, which showed that depression levels increased starting in the first year of study, peaked in the third year, and then decreased sharply in the final year before graduation [73]. However, another study has indicated that senior students are more likely to experience depression than undergraduates, as they face various new stressors during their graduation year, which is a crucial time for them to continue their education or enter the workforce [74].

Another contributing factor to the increased prevalence of depression is that college students who have previously failed exams are more likely to experience depression because they may feel guilty or inadequate, which can lead to feelings of depression. This study found a strong correlation between depression, suicidal ideation, and academic stress, particularly subpar performance. Higher psychological discomfort was associated with depressive symptoms and suicidal ideation among students who struggled academically. According to this link, students’ mental health is significantly impacted by academic demands such as performance issues and career worry. These results emphasise the importance of colleges acknowledging the psychological effects of academic difficulties and offering coping mechanisms to students.

These findings are supported by Chemagosi [86], who emphasized the important role that academic pressure plays in students’ well-being, and Soh [87], who talked about how academic stress causes psychological suffering and influences students’ behavior while seeking aid, both support these findings. According to Alotaibi et al. [88], to lessen the impact of psychological diseases on students, higher education institutions must provide sufficient counselling services. This finding highlights the need for a support network. Further demonstrating the significance of addressing mental health in educational contexts, Karimah and Robin [89] investigated the impact of academic stress on psychological well-being.

Students with a family history of mental illness may also be at greater risk due to environmental or genetic factors. Even though most students reported being financially stable, those from larger homes or those coping with parental separation felt more emotional stress, which could worsen mental health issues. Male and female students’ depression levels did not differ considerably, suggesting that depression was experienced similarly by both genders in this situation.

The findings related to family demographic data and their association with students’ depression revealed that students with older parents and parents with lower levels of education were more likely to experience higher depression levels. This may be attributed to the additional responsibilities that students bear in caring for their aging parents, which can create emotional and physical burdens for them. Furthermore, the difficulty in effectively communicating university-related stressors to illiterate parents due to differing levels of understanding may hinder students’ ability to express and process their negative emotions, exacerbating feelings of isolation and stress.

This aligns with the study conducted in Ethiopia by Ahmed et al. [90], who found that most participants’ parents had low levels of education. This could lead to students with low parental educational status feeling less confident and experience greater psychological strain.

Additionally, parental marital status emerged as a significant factor contributing to student depression. The findings indicate a higher prevalence of depression among students with divorced parents. This could be attributed to the negative impact of a disrupted family environment, which often results in emotional instability and a diminished sense of security. The lack of a strong and supportive family structure might make students feel even more alone and stressed because they do not have a solid foundation to help them deal with the demands of college life. These elements highlight the importance of a stable and supportive family structure in fostering students’ resilience and mental health. These findings are consistent with Pan et al. [91], who found that marital relationship negatively predicted adolescents’ depressive symptoms.

### Limitations of the study

This study has several limitations. Firstly, the cross-sectional design of the study limits the ability to establish causality between parenting styles and mental health outcomes. Second, is this study’s reliance on self-reported data, which may introduce response and recall biases, particularly concerning sensitive topics such as depression and suicidal ideation. Furthermore, the lack of proportional sampling based on estimated student population numbers for each faculty, instead opting for fixed numbers from each faculty, may affect the generalizability of the findings to the entire university. Lastly, the focus on a single university in Egypt may diminish the external validity of the results for other student populations or cultural contexts. Future longitudinal studies with more participants from various other regions and universities are recommended to enhance the generalizability of these findings.

### Conclusion and recommendation

This study concluded that parenting styles, especially the authoritative style, significantly impact the mental well-being of university students and are linked to reduced depression and suicidal thoughts. Depression acts as a key intermediary between parenting style and suicidal tendencies, with both mothers and fathers having substantial influence. These results highlight the need to encourage supportive parenting methods and ensure the availability of mental health resources in university environments. Policymakers and university leaders should develop culturally sensitive programs to enhance mental health and encourage parental involvement.

Based on the findings of this study, several recommendations have been identified to support college students experiencing mental health challenges. Firstly, it is essential to provide on-campus counseling services staffed by qualified professionals. Secondly, mentors should receive training to recognize signs of student distress and report them appropriately. Thirdly, enhancing the mental health literacy of college students is crucial. Lastly, it is important to conduct awareness programs for parents at primary healthcare facilities, focusing on the impact of parenting styles on the mental health of children and adolescents, as well as promoting optimal parenting practices to support their mental well-being.

### Relevance for clinical practice and research

The present results highlights the critical impact of factors associated with parenting styles, thus linking the authoritative style as essential for reducing depression and suicidal ideation. These findings are not true in everyday practice. The integration of information provided by family dynamics requires critical attention during assessment or as an intervention strategy in clinical practice. In essence, parental education on warmth and structured support ensures good psychological outcomes for children. Such a study provides opportunities for clarity or explanation of country-specific parenting methods that can be used to understand the long-term mental health impacts. This may also provide opportunities for integrating parenting education into extensive common mental health promotion initiatives targeting higher-education students as an option for interdisciplinary studies.

## Data Availability

No datasets were generated or analysed during the current study.
